# The effectiveness and associated factors of online psychotherapy on COVID-19 related distress: A systematic review and meta-analysis

**DOI:** 10.3389/fpsyg.2022.1045400

**Published:** 2022-11-09

**Authors:** Danni Chi, Yuanyuan Zhang, Dongsheng Zhou, Guozhang Xu, Guolin Bian

**Affiliations:** ^1^Ningbo Key Laboratory of Sleep Medicine, Ningbo Kangning Hospital, Affiliated Tongyi Hospital of Medical College of Ningbo University, Ningbo, China; ^2^Ningbo Municipal Center for Disease Control and Prevention, Ningbo, China

**Keywords:** psychotherapy, COVID-19, online, effectiveness, setting, telepsychology, telepsychiatry

## Abstract

**Objective:**

A quantitative synthesis of online psychotherapies' effectiveness in managing COVID-19 related distress is lacking. This study aimed to estimate online psychological interventions' effectiveness and associated factors on COVID-19 related psychological distress.

**Methods:**

Multi-databases including PubMed, EBSCO, ProQuest, and Cochrane were searched repeatedly till the end of June 2022. Hand-picking was also utilized for relevant papers. Depression, anxiety, stress, and quality of sleep were evaluated as outcomes. The risk of bias was evaluated using the Cochrane tool. Data analyses were conducted through Review Manager (version 5.4.1).

**Results:**

A total of 13 studies involving 1,897 participants were included for meta-analysis. Results showed that online psychotherapy significantly reduced the levels of depression [standard mean difference, SMD = −0.45, 95% CI (−0.69, −0.20)], anxiety [SMD = −0.67, 95% CI (−0.99, −0.36)], and stress [SMD = −0.73, 95% CI (−1.11, −0.34)], but not quality of sleep [SMD = −0.53, 95% CI (−1.23, 0.17)]. In addition, guided therapies were more effective than self-help ones on reducing levels of anxiety (χ^2^ = 5.58, *p* = 0.02, and *I*^2^ = 82.1%), and ≤ 2 weeks' daily interventions were more effective on treating depression than 2-month weekly interventions (χ^2^ = 7.97, *p* = 0.005, *I*^2^ = 87.5%).

**Conclusion:**

Online psychological interventions effectively reduced COVID-19 related depression, anxiety, and stress levels, and the effectiveness was influenced by settings like guidance and duration and frequency.

**Systematic review registration:**

https://inplasy.com/inplasy-2022-7-0081/, identifier: INPLASY202270081.

## Introduction

The coronavirus disease 2019 (COVID-19) pandemic has affected every aspect of people's lives worldwide. There have been 553 million confirmed COVID-19 cases and more than 6.3 million deaths globally (World Health Organization, 2022). It raises a constellation of issues which challenges people's mental health, including social isolation, worries of infection of the self and significant others, financial difficulties, and uncertainties (Brooks et al., 2020). More psychosomatic problems or exacerbations of psychiatric symptoms were found in people with COVID-19 and preexisting mental illness, healthcare workers, and the general population (Vindegaard and Benros, 2020). Thus, effective mental health services are essential and urgent under such a circumstance. In the post-pandemic era, online psychological interventions have been boosted and broadly accepted, as it is more accessible and efficient than traditional onsite or face-to-face psychotherapy (Wind et al., 2020).

The COVID-19 related distress refers to a broad range of mental experiences and symptoms related with the COVID-19 pandemic, with or without clinical diagnoses. Among these COVID-19 related distress and psychiatric symptoms, depression, anxiety, sleeping disorder, and stress-related symptoms have been mostly reported (Brooks et al., 2020; Salari et al., 2020; Vindegaard and Benros, 2020). Due to the urgent and extensive need for online psychological interventions during the pandemic, online psychological interventions have been delivered in various forms and settings. Online interventions, for example, single-session mindfulness, 1-week psychoeducational support, 4-session weekly CBT, and 8-week daily mindfulness have been reported to be effective in treating COVID-19 related distress (Farris et al., 2021; Mahoney et al., 2021; Mirhosseini et al., 2021; Nourian et al., 2021). However, many of these studies were not conducted in randomized controlled trials (e.g., without control groups). The effectiveness and associated factors of online psychological interventions on COVID-19 related distress have yet to be clarified. Therefore, this meta-analysis aimed to estimate the effectiveness of online psychological intervention of COVID-19 related distress in randomized controlled trials and explore associated influential factors.

## Materials and methods

This study was conducted according to the preferred reporting items for systematic review and meta-analyses (PRISMA, Moher et al., 2009).

### Search strategy

This review followed the established guidelines of evidence-based clinical review to ensure clarity and transparency. The topic was relevant and of common clinical interest. A systematic literature search was initially conducted in April 2022 and re-ran to identify newly published studies on 24 June 2022. Multi-databases were searched as suggested (Siwek et al., 2002), including PubMed, EBSCO, ProQuest, and Cochrane (Table 1). Hand-picked processes were also applied to find the references in relevant articles and reviews. In addition, emails were sent to authors who registered potentially relevant protocols to detect completed but not yet published papers.

**Table 1:** Search queries.

**Database**	**Queries**	**Hits**
PubMed	(((“online”[Title] OR “remote”[Title] OR “internet”[Title]) AND (“psychological”[Title/Abstract])) AND (“intervention”[Title/Abstract] OR “treatment”[Title/Abstract] OR “counsel*”[Title/Abstract] OR “therap*”[Title/Abstract])) AND (“COVID-19”[Title/Abstract] OR “coronavirus”[Title/Abstract] OR “2019-ncov”or “SARS-CoV-2”[Title/Abstract] OR “cov-19”[Title])	132
EBSCO	TI (“online” or “internet” or “remote”) AND TI (“COVID-19” or “coronavirus” or “2019-ncov” or “SARS-CoV-2” or “cov-19”) AND SU (“therap*” or “counsel*” or “intervention” or “treatment”) AND SU (psycho*)	132
ProQuest	Abstract(“online” or “internet” or “remote”) AND Abstract(“COVID-19” or “coronavirus” or “2019-ncov” or “SARS-CoV-2” or “cov-19”) AND Abstract(“therap*” or “counsel*” or “intervention” or “treatment”) AND Abstract(psycho*)	54
Cochrane	(online OR remote OR internet):ti,ab,kw AND (“COVID-19” or “coronavirus” or “2019-ncov” or “SARS-CoV-2” or “cov-19”):ti,ab,kw AND (“therap*” or “counsel*” or “intervention” or “treatment”):ti,ab,kw AND (psycho):ti,ab,kw (Word variations have been searched)	13
Hand-picked	Though reading related articles and reviews	8
Email	Three emails were sent to authors who published relevant protocols and might have unpublished papers, but none respond	0

### Study selection

After the removal of studies of non-adult participants, not written in English, and duplicates, titles and abstracts were screened. Two authors (DC and YZ) independently screened the lists of titles/abstracts identified through database searching according to inclusion and exclusion criteria. Any discrepancies were resolved by discussions with a third member of the team (DZ). Then, the potentially relevant records were retrieved in full text for eligibility checking.

This study used relatively broad inclusion criteria as it had been only more than 2 years since the COVID-19 pandemic. We included studies that:

- used validated quantitative measures to examine the effects of the interventions on depression, anxiety, perceived psychological stress or distress, and quality of sleep;
- administered interventions to people aged 18 years old or above;
- delivered psychological interventions online through digital devices;
- used a randomized controlled design;
- administered to people who perceived distress related to COVID-19.

The exclusion criteria included:

- The intervention was not well-supported by empirical evidence;
- The intervention was not aimed at treating COVID-19 related psychological conditions;
- Participants in studies were patients with preexisting mental or severe physical illnesses;
- The article did not provide sufficient data to calculate the effect sizes.

### Data extraction

Data extraction was conducted by the first author (DC) and checked by another author (DZ). Disagreements were resolved by discussion. Extracted data included: first author, year and country of publication, population, characteristics of populations (e.g., age and percentage of female), characteristics of intervention (type, guidance, delivery mode, sessions and durations), sample sizes of treated and control groups, and outcome measures (e.g., depression, anxiety, stress, and quality of sleep). Two authors were contacted and requested for extra data with regard to the data extraction. However, neither of them responded.

### Risk of bias assessment

The risk of bias assessment was conducted by GX and GB independently based on Cochrane's suggestions (Higgins and Altman, 2008), and discrepancies were resolved through discussion. Criteria provided in Review Manager (version 5.4.1) were applied: random sequence generation and allocation concealment, blinding of participants and outcomes assessment, incomplete outcome data, selective reporting, and other biases. Each criterion was assessed as “low risk”, “unclear”, or “high risk”. The overall quality was considered as “high” if all criteria were assessed as low risk of bias, as “low” if one or more criteria were assessed as high risk of bias, and as “moderate” if they did not fit the first two situations.

### Statistical analyses

Data were analyzed with Cochrane RevMan (version 5.4.1). The effect of online psychological intervention compared to inactive control was assessed using the standardized mean difference (SMD) at post-treatment as the outcome. For each comparison between a treated and a control group, effect sizes were calculated per outcome variable (i.e., depression, anxiety, stress, and quality of sleep). If more than one instrument were used to measure the same outcome, the more valid and commonly used one was adopted. One study used the total score rather than the subscales of DASS to measure general psychological distress (Brog et al., 2022), and thus, this total score was considered as a measure of stress. All outcomes were continuous variables in this meta-analysis, and SMD with a 95% confidence interval (CI) were used to present the pooled results. Heterogeneity was assessed with χ^2^ and *I*^2^ (Higgins and Thompson, 2002). The random-effects model was used as variations across studies are inevitable in real settings. Forest plots were used to assess variations in effects across studies. Sensitivity analyses were conducted to test the stability of the results by assessing whether study quality was related to outcome by comparing the low-risk studies in risk of bias assessment. Funnel plots were used to assess the publication bias. The level of significance in this study was set to *p* < 0.05.

## Results

### Study selection

**Figure 1:**
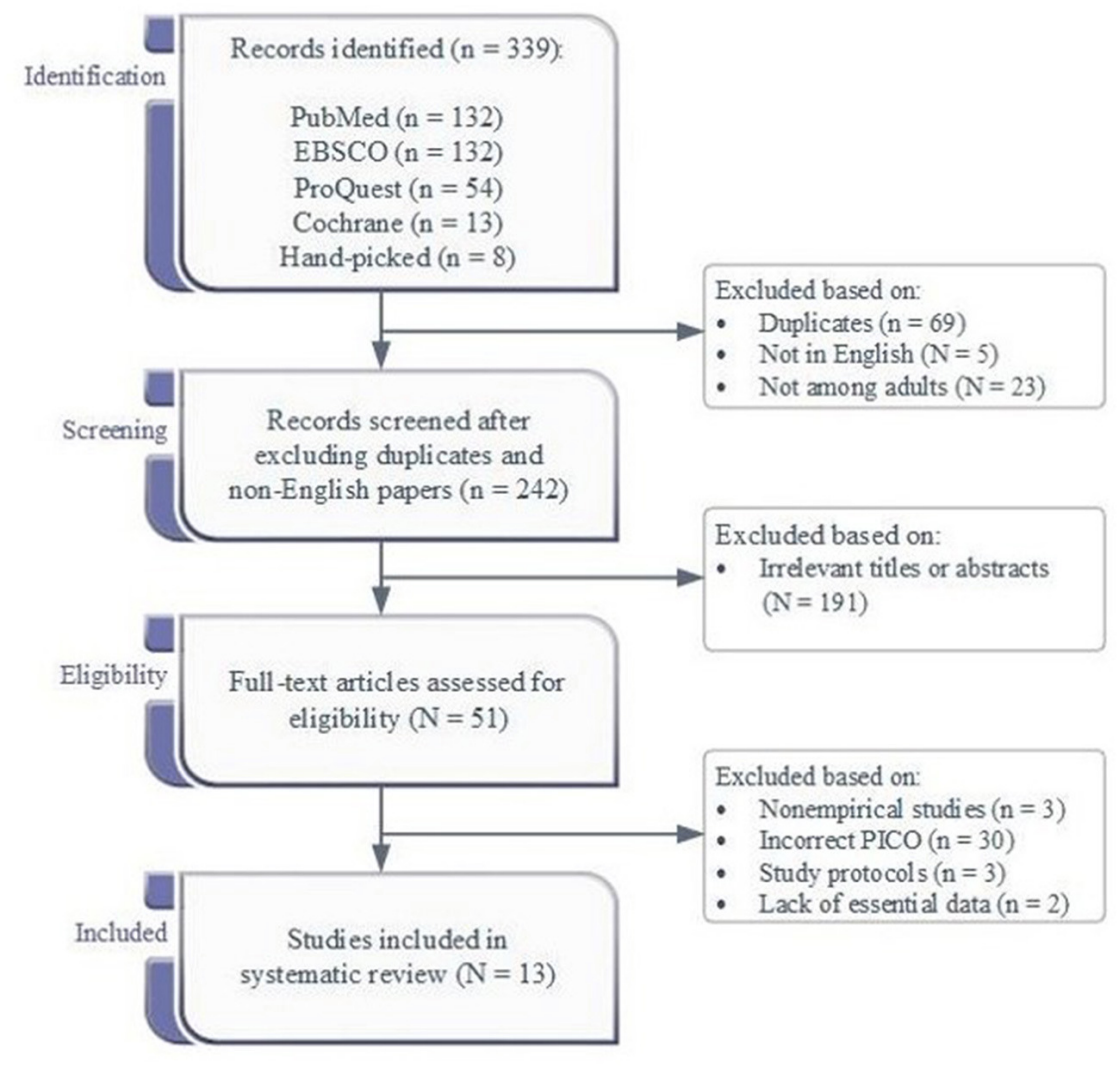
The process diagrammatically study selection.

Figure 1 presented the flow diagram of the study selection process. The database searches and hand-picking produced 339 records. After excluding the duplicates and studies not written in English or conducted in adults, 242 records were screened, and 191 were excluded due to irrelevant titles or abstracts. Of the 51 full-text articles checked for eligibility, 15 fit the criteria. However, two of them were lack of essential data. Emails were sent to the authors to request relevant data, but the authors did not respond. In addition, no unpublished data were available after sending emails to three authors who had published potentially relevant protocols. Thus, 13 records were included.

### Description of included studies

The included studies were conducted in many different countries, and Table 2 summarized their characteristics.

**Table 2:** Characteristics of studies included in the meta-analysis.

**References**	**Country** **population**	**F%^a^**	**Mean age** **(range)**	**Intervention** **(*n*)**	**Guidance**	**Delivery** **mode**	**Frequency** **(durations)**	**Control** **(*n*)**	**Data** **collection**	**Outcomes** **measurements**
Al-Refae et al. (2021)	Canada, general	79	25.24 (18–66)	MCBT (78)	Self-help	Mobile app	Weekly (4 weeks)	87	Pre, post	DASS-depression, anxiety, stress
Aminoff et al. (2021)	United States, general	71	42.7 (21–78)	CBT (20)	Therapist	Online platform	Weekly, (7 weeks)	23	Pre, post	PHQ-9, GAD-7, PSS, ISI
Brog et al. (2022)	Switzerland, general	81	40.36 (18–81)	CBT-PPI (45)	Self-help	Website	Twice per week, (3 weeks)	52	Pre, post, follow up	PHQ-9, DASS total
Carbone et al. (2022)	Italy, general	77	34.34 (≥18)	CBT (26)	Therapist	Online platform	Single session	27	Pre, post	STICSA, PANAS-N
Khah et al. (2021)	Iran, COVID patients	50	32.5 (20–45)	Mindfulness (20)	Self-help	Website	Twice per day (2 weeks)	20	Pre, post	BDI-II, PSS
Krifa et al. (2021)	Tunis, undergraduates	94	20.74 (18–25)	Mindfulness-PPI (159)	Self-help	Website	Weekly (8 weeks)	165	Pre, post, follow up	DASS-depression, anxiety, stress
Liu et al. (2020)	China, undergraduates	45	50.41 (≥20)	Muscle relaxation (25)	Therapist	Call center	Daily (5 days)	26	Pre, post	STAI, SRSS
MacDonald and Neville (2022)	United States, undergraduates	89	20.78 (19–25)	Mindfulness (17)	Therapist	Online platform	Weekly (8 weeks)	10	Pre, post	DASS-depression, anxiety, stress
Nourian et al. (2021)	Iran, HC workers	83	35.6 (≥18)	Mindfulness (19)	Therapist	Mobile app	Daily (8 weeks)	22	Pre, post	PSQI
Otared et al. (2021)	Germany, HC workers	45	about 32 (≥ 18)	ACT (20)	Therapist	Online platform	Weekly (8 weeks)	20	Pre, post	BDI-II, BAI
Rackoff et al. (2022)	United States, undergraduates	72	20.24 (18–24)	CBT (165)	Self-help	Website	Almost daily (4 weeks)	204	Pre, post, follow up	DASS-depression, anxiety, stress
Shaygan et al. (2021)	Iran, Covid patients	44	36.77 (≥ 18)	Mindfulness & CBT (26)	Self-help	Mobile app	Daily (2 weeks)	22	Pre, post	PSS
Wahlund et al. (2021)	Sweden, general	80	about 46 (18–81)	CBT (285)	Self-help	Website	Twice per week (3 weeks)	314	Pre, post, follow up	MADRS-S, GAD-7, ISI

Only measurements that used in the meta-analysis were reported. HC, health care; ACT, acceptance and commitment therapy; CBT, cognitive behavioral therapy; MBCT, mindfulness based cognitive therapy; DASS, Depression Anxiety and Stress Scale; PHQ-9, Patient Health Questionnaire 9-item scale; GAD-7, Generalized Anxiety Disorder 7-item scale; PSS, Perceived Stress Scale; ISI, Insomnia Severity Index; STICSA, State-Trait Inventory for Cognitive and Somatic Anxiety; PANAS-N, Positive and Negative Affect Schedule–Negative subscale; BDI-II, Beck Depression Inventory-II; STAI, State-Trait Anxiety Scale; SRSS, Sleep State Self-Rating Scale; PSQI, Pittsburgh Sleep Quality Index; BAI, Beck Anxiety Inventory; MADRS-S, Montgomery Asberg Depression Rating Scale-Self rated.

a F% : percentage of female.

#### Population characteristics

The total population comprised 1,897 participants, of which 905 were in the treated groups and 992 were in the control groups. All participants were adults, with a mean age ranging from 20.24 to 50.41 years. The majority of the sample was female in nine of the included studies. The total sample size ranged from 27 (MacDonald and Neville, 2022) to 599 (Wahlund et al., 2021). All included studies were conducted in populations without previously identified mental or severe physical illnesses. Three studies were conducted among COVID-19 patients with mild symptoms (Liu et al., 2020; Khah et al., 2021; Shaygan et al., 2021), two were in health care workers (Nourian et al., 2021; Otared et al., 2021), three were in undergraduates (Krifa et al., 2021; MacDonald and Neville, 2022; Rackoff et al., 2022), and others were in the general population (Al-Refae et al., 2021; Aminoff et al., 2021; Brog et al., 2022; Carbone et al., 2022).

#### Intervention

The online interventions reviewed in this study aimed to reduce COVID-19 related psychological distress and/or promote participants' mental health. CBT (*n* = 5) and mindfulness (*n* = 4) were mostly adopted interventions, and two studies used integrated interventions of CBT and mindfulness, and the other two used ACT (Otared et al., 2021) and progressive muscle relaxation (Liu et al., 2020), respectively. The interventions were either therapist-guided (*n* = 6) or self-helped (*n* = 7) in which participants were provided with access to online materials. The interventions were delivered through online platforms like Zoom (*n* = 4), websites (*n* = 5), other mobile applications (*n* = 3), and hospital's call system (*n* = 1). Frequencies in these studies were daily (*n* = 5), twice per week (*n* = 2), weekly (*n* = 5), or a single session (*n* = 1), and the duration of interventions ranged from a single session to 8 weeks.

#### Comparison

Each of the 13 studies used an inactive control. Studies in COVID-19 patients provided routine care to control groups (Liu et al., 2020; Khah et al., 2021; Shaygan et al., 2021). Treated groups were compared with waitlist control groups (*n* = 10) or routine care groups (*n* = 3). One study provided music files and materials for caring to the control group, whereas the treated group was given guided mindfulness training (Nourian et al., 2021). Other studies used waitlist groups as inactive control.

#### Outcomes

All studies measured the outcomes pre- and post-intervention. Four studies reported follow-up data but were not analyzed in this study. The numbers of studies which measured depression, anxiety, stress, and quality of sleep were 9, 9, 9, and 4, respectively.

#### Risk of bias assessment

**Figure 2:**
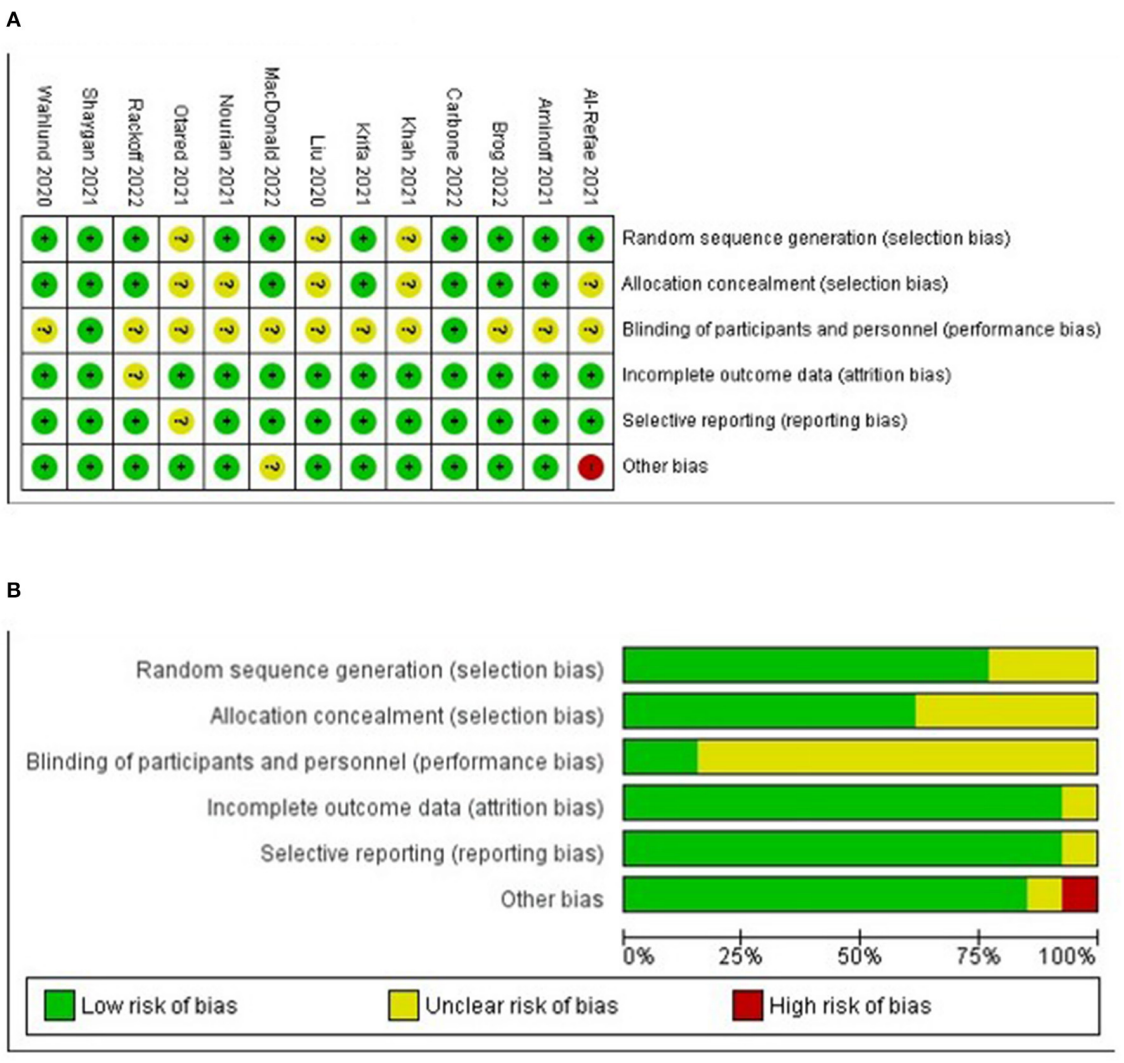
**(A)** Risk of bias summary. **(B)** Risk of bias graph.

In studies that used self-reporting, the criterion of blinding outcomes assessment was not applicable. Therefore, six criteria rather than seven were utilized. Many studies used waitlist control, and the criterion of blinding intervention to participants was not met. The risk of bias assessment showed that most studies were of high (*n* = 2) or moderate (*n* = 11) quality. One study was assessed as low quality due to conflicts of interest: Two of the authors were the co-founders of the mobile application used in their study, and the application was going to be commercialized (Al-Refae et al., 2021). Figures 2A,B present the risk of bias summary and graph, respectively.

### Meta-analysis

#### Overall effects

The between-group effects at post-intervention for depression, anxiety, stress, and sleep are presented in Table 3, and Forest plots are shown in Supplementary Figure S1.

**Table 3:** Comparative effectiveness between treated and control group in terms of depression, anxiety, stress, and quality of sleep.

**Outcome**	**N_comp_**	**SMD**		**Overall effect**		**Heterogeneity**			
		**IV**	**95% CI**	***Z***	***p***	**τ^2^**	**χ^2^**	***p***	***I*** **^2^**
Depression	9	−0.45	(−0.69, −0.20)	3.57	<0.001	0.09	37.11	<0.001	78%
Anxiety	9	−0.67	(−0.99, −0.36)	4.16	<0.001	0.17	60.14	<0.001	87%
Stress	9	−0.73	(−1.11, −0.34)	3.70	<0.001	0.28	65.09	<0.001	88%
Sleep	4	−0.53	(−1.23,0.17)	1.47	0.14	0.43	25.61	<0.001	88%

CI, confidence interval; SMD, standard mean difference; N_comp_, number of comparisons.

A total of nine studies reported levels of pre- and post-treatment depression. A significant effect of online psychotherapy on depression was observed: SMD = −0.45 and 95% CI = (−0.69, −0.20), and the heterogeneity was also significant: τ^2^ = 0.09, χ^2^ = 37.11, *p* < 0.001, and *I*^2^ = 78%.

Based on the nine comparisons, a significant effect of online psychological interventions on anxiety was observed: SMD = −0.67, 95% CI = (−0.99, −0.36). A significant heterogeneity was also observed: τ^2^ = 0.17, χ^2^ = 60.14, *p* < 0.001, and *I*^2^ = 87%.

The significant overall effect size for nine comparisons on perceived stress was observed: SMD = −0.73 and 95% CI = (−1.11, −0.34), with a significant level of heterogeneity (τ^2^ = 0.28, χ^2^ = 65.09, *p* < 0.001, and *I*^2^ = 88%).

Based on the four studies, the effect of online psychological interventions on quality of sleep was not significant, SMD = −0.53 and 95% CI = (−1.23, 0.17), with a significant heterogeneity (τ^2^ = 0.43, χ^2^ = 25.61, *p* < 0.001, and *I*^2^ = 88%).

#### Subgroup analyses

Exploratory subgroup analyses were summarized in Table 4. Criterions included types of intervention (CBT or mindfulness), guidance (therapist-guided or self-help), and duration and frequency ( ≤2 weeks daily vs. 2-month weekly). Subgroup analyses were conducted to explore the effect differences of online psychological intervention on depression, anxiety, and stress, but not the quality of sleep due to limited studies (*n* = 4).

**Table 4:** Effect differences between subgroups in terms of depression, anxiety, and stress.

**Outcome**	**Criterion**	**Subgroups**	***N*** **_comp_**	**Standard mean difference**		**Overall effect**		**Heterogeneity (*I^2^*)**	**Subgroup differences**		
				**IV**	**95% CI**	***Z***	***p***		***χ*** ***^2^***	***p***	***I*** **^2^**
Depression	Type	CBT	4	−0.20	(−0.40, −0.00)	2.00	0.05	50%	4.00	0.05	75.0%
		Mindfulness	3	−0.93	(−1.61, −0.24)	2.65	0.008	76%			
	Guidance	Therapist	3	−0.50	(−0.88, −0.11)	2.54	0.01	0	0.05	0.82	0
		Self-help	6	−0.44	(−0.74, −0.14)	2.91	0.004	86%			
	Duration & frequency	≤ 2 weeks, daily	1	−1.78	(−2.53, −1.04)	4.70	<0.001	NA	7.97	0.005	87.5%
		7–8 weeks, weekly	4	−0.68	(−0.87, −0.48)	6.81	<0.001	0			
Anxiety	Type	CBT	4	−0.50	(−0.95, −0.06)	2.23	0.03	88%	0.29	0.59	0
		Mindfulness	2	−0.64	(−0.86, −0.42)	5.83	<0.001	0			
	Guidance	Therapist	5	−1.16	(−1.75, −0.57)	3.86	<0.001	74%	5.58	0.02	82.1%
		Self-help	4	−0.35	(−0.67, −0.03)	2.12	0.03	88%			
	Duration and frequency	≤ 2 weeks, daily	1	−1.09	(−1.68, −0.49)	3.60	<0.001	NA	0.01	0.92	0
		7–8 weeks, weekly	4	−1.04	(−1.75, −0.32)	2.85	0.004	84%			
Stress	Type	CBT	4	−0.51	(−1.11,0.09)	1.67	0.09	88%	1.88	0.17	46.8%
		Mindfulness	3	−1.18	(−1.93, −0.43)	3.10	0.002	78%			
	Guidance	Therapist	3	−0.99	(−1.62, −0.35)	3.04	0.002	62%	0.85	0.36	0
		Self-help	6	−0.62	(−1.07, −0.17)	2.69	0.007	90%			
	Duration & frequency	≤ 2 weeks, daily	2	−1.42	(−2.76, −0.08)	2.07	0.04	86%	0.65	0.42	0
		7–8 weeks, weekly	3	−0.86	(−1.07, −0.65)	8.12	<0.001	0			

N_comp_, number of comparisons; CBT, cognitive behavioral therapy.

For depression, significant larger effect was found in shorter and intense sessions (i.e., daily and ≤ 2 weeks): χ^2^ = 7.97, *p* = 0.005, and *I*^2^ = 87.5%. Effect differences on depression among other subgroups were insignificant (Table 4). For anxiety, larger effect was found in therapist-guided interventions: χ^2^ = 5.58, *p* = 0.02, and *I*^2^ = 82.1%. None of the effect differences on stress between subgroups was significant (Table 4).

### Publication bias and sensitivity analysis

Funnel plots for each outcome were presented in Supplementary Figure S2. No significant publication bias was identified. Leave-one-out sensitivity analysis was conducted, and no single study had a substantial influence on the overall effect sizes.

## Discussion

### Main findings

This meta-analysis included 13 studies and confirmed the overall effects of online psychological interventions in reducing COVID-19 related depression, anxiety, and stress, and explored associated factors that might influence the effectiveness, such as types of intervention (CBT or mindfulness), guidance (therapist involved or self-help), and duration and frequency ( ≤2 weeks daily or 2-month weekly).

This present study found that CBT and mindfulness were the most commonly used online interventions among the 13 included studies. The effectiveness of online mindfulness has also been well-supported in treating general mental conditions and COVID-19 related distress (Spijkerman et al., 2016; Wright et al., 2019; Yeun and Kim, 2022). Online guided CBT was found as effective as face-to-face CBT in treating mental and somatic conditions in a meta-analysis that reviewed 20 studies (Carlbring et al., 2018), though it is not specific to the pandemic. No published studies have compared the effectiveness between online CBT and mindfulness on COVID-19 related distress, and this present study found non-significant differences in effectiveness between them in treating COVID-related depression, anxiety, and stress.

The findings of this study suggested that both guided and online self-help interventions were effective in managing the pandemic related depression, anxiety, and stress, and that therapist-guided interventions were more effective than self-help ones in treating COVID-19 related anxiety, but not depression or stress. In partially consistent with the findings of this present study, a recent study found that though both therapist-guided and self-help online 6-week CBT treatment were effective in reducing COVID-19 related depression and anxiety levels, therapist-guided was more effective than the self-help one (Al-Alawi et al., 2021). Similarly, the superiority of guided online treatment (i.e., mindfulness-based interventions) in managing depression, anxiety, stress, and wellbeing in general and clinical populations was also reported in two meta-analyses (Spijkerman et al., 2016; Wright et al., 2019; Zhang et al., 2020). However, A four-session self-help CBT has been found to have similar effects to the clinician-guided CBT in reducing levels of depression and anxiety in young adults at post-treatment and 3- and 12-month follow-ups (Dear et al., 2018). It is possible that factors like patients' adherence and clinicians' estimation of treatment response influenced the effectiveness of online interventions (Salomonsson et al., 2020), but both of them compromised in self-help treatment, and so did the effectiveness.

The findings of this study suggested that intense and short-term interventions (i.e., ≤2 weeks' daily sessions) were more effective in reducing depression than weekly and longer ones (i.e., 7–8 weeks). Short-term daily sessions might be more effective and efficient than 2-month weekly ones in treating COVID-19 related depression, though the long-term effects of these two types of sessions were not clear. Though limited published studies investigated the impact of frequency and duration on the effectiveness of COVID-19 studies, studies have found that the number of total sessions moderated the effectiveness of online mindfulness-based interventions in treating stress (Spijkerman et al., 2016). However, as most studies reviewed in Spijkerman et al.'s meta-analysis were conducted weekly, and thus the potential moderator role of frequency as unable to explore.

### Limitations and implications

In the post-pandemic era, enormous needs for psychological interventions are yet to be fulfilled. Online psychological intervention can benefit a broad range of people with lower costs and higher accessibility without worrying about exposure to the infection (Wind et al., 2020). However, few studies reviewed online psychological interventions of COVID-19 related distress, and the effective settings are yet to be clarified. This study is of the few (if not none) to confirm the effectiveness of online psychological interventions in reducing COVID-19 related distress (e.g., depression, anxiety, sleeping disorder, and stress) and identify associated factors. These findings can help practitioners and therapists set and deliver more effective and efficient online psychological interventions to people experiencing COVID-19 related distress.

There were some limitations of this study. Included studies used waitlist control or routine care control for COVID-19 patients, which means the effectiveness of online psychological interventions was based on comparisons with those who did not receive psychological support or interventions. It was unclear whether online psychological interventions were more effective than psychoeducation or psychological support groups. Considerable variability in online psychological settings existed, and the limited sample size constrained us from further exploring the interactions of these different settings.

Future studies need to use more active control (e.g., online psychoeducation or online psychological support) to investigate the effectiveness of online psychotherapy. Appropriate settings can promote effectiveness. As a relatively new mode of delivering psychological interventions, empirical studies on identifying more effective and efficient settings are needed. Future studies need to investigate the stability of online psychological interventions, and a more structured guideline can benefit people globally in the post-pandemic era.

Clinicians or psychotherapists can involve in the online psychological interventions or some of the sessions. Self-help interventions are better than no interventions at all, and the effectiveness can be enhanced with the involvement or guidance of therapists. Moreover, in treating with COVID-19 related psychological distress, people are more likely to benefit from daily and short-term sessions (i.e., ≤2 weeks) rather than weekly and longer treatment (i.e., 7–8 weeks). Therefore, clinicians and psychotherapists are encouraged to use online psychological interventions as an effective and accessible way to deliver service in the post-pandemic era.

## Conclusion

This study estimated the effectiveness and explored the influential factors of online psychological interventions on COVID-19 related distress. The findings confirmed the effectiveness of online psychological interventions in relieving the COVID-19 related depression, anxiety, and perceived stress, but not in improving sleeping quality. Settings like with or without guidance and frequency and duration also influenced the effectiveness of the online psychological interventions. Online psychological interventions with appropriate settings are required to benefit more people in but not limited to the COVID-19 post-pandemic era.

## Data availability statement

The original contributions presented in the study are included in the article/Supplementary material, further inquiries can be directed to the corresponding authors.

## Author contributions

DC, YZ, and DZ designed the searching queries, searched the databases, screened the records, and checked the eligibility of the records. DC and DZ contributed to the data extraction, analyses, and draft writing. GX and GB contributed to the supervision of the process, assessing the bias, and reviewing and editing the draft. All authors contributed to the conceptualization of the study. All authors agree to be accountable for the content of the work and approved the submitted version.

## Funding

The study was supported by the Ningbo Municipal Emergency Science and Technology Major Project (No. 2022Z034) and the Medical and Health Brand Discipline in Ningbo (No. PPXK2018-08).

## Conflict of interest

The authors declare that the research was conducted in the absence of any commercial or financial relationships that could be construed as a potential conflict of interest.

## Publisher's note

All claims expressed in this article are solely those of the authors and do not necessarily represent those of their affiliated organizations, or those of the publisher, the editors and the reviewers. Any product that may be evaluated in this article, or claim that may be made by its manufacturer, is not guaranteed or endorsed by the publisher.
