# Supplementary figures and images for: The effectiveness and associated factors of online psychotherapy on COVID-19 related distress: A systematic review and meta-analysis
Source: Front Psychol. 2022 Nov 9;13:1045400. doi: 10.3389/fpsyg.2022.1045400 (PMC9682141; doi:10.3389/fpsyg.2022.1045400)

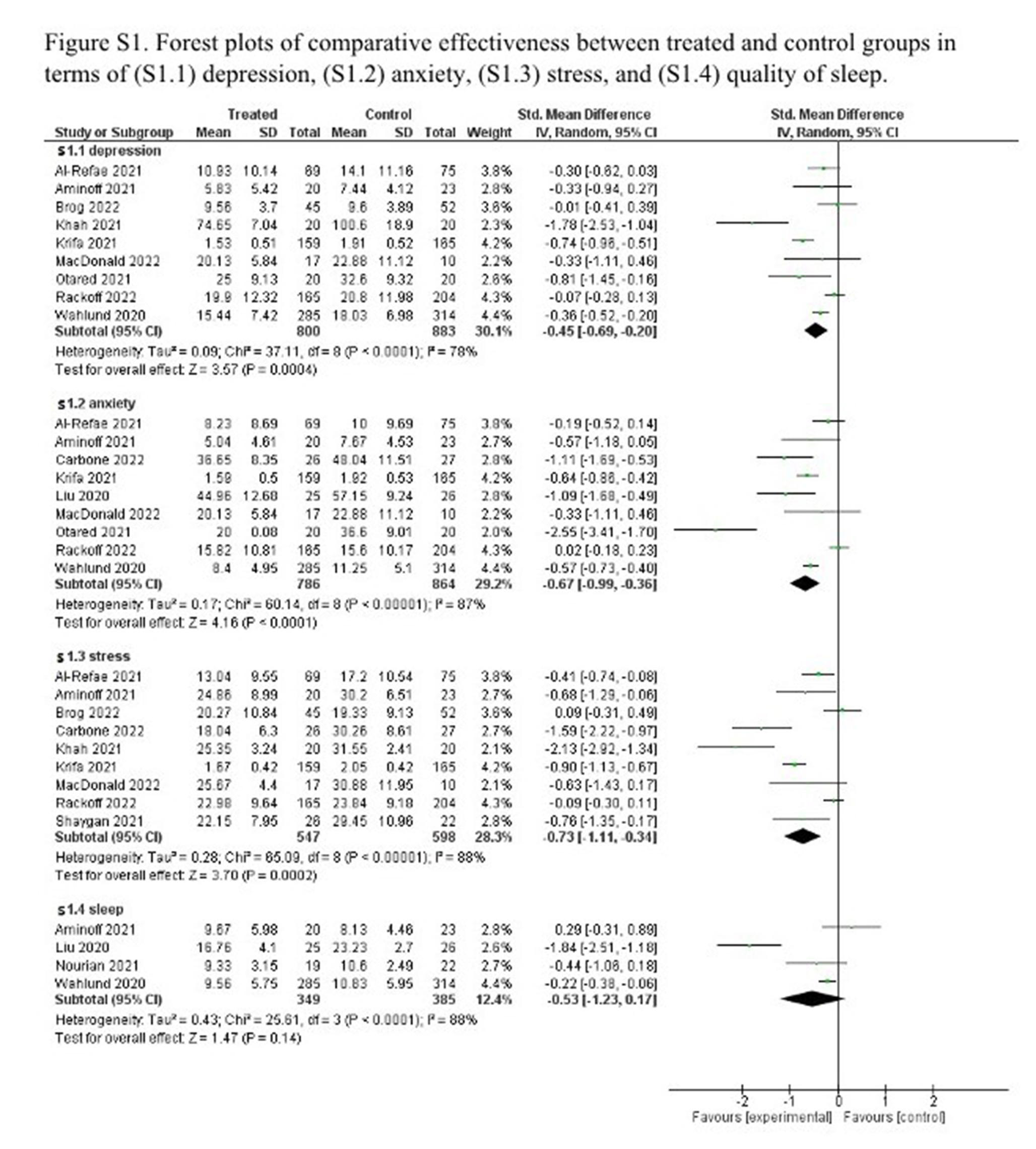

Supplement: Supplementary file 1 [file Image_1.JPEG]

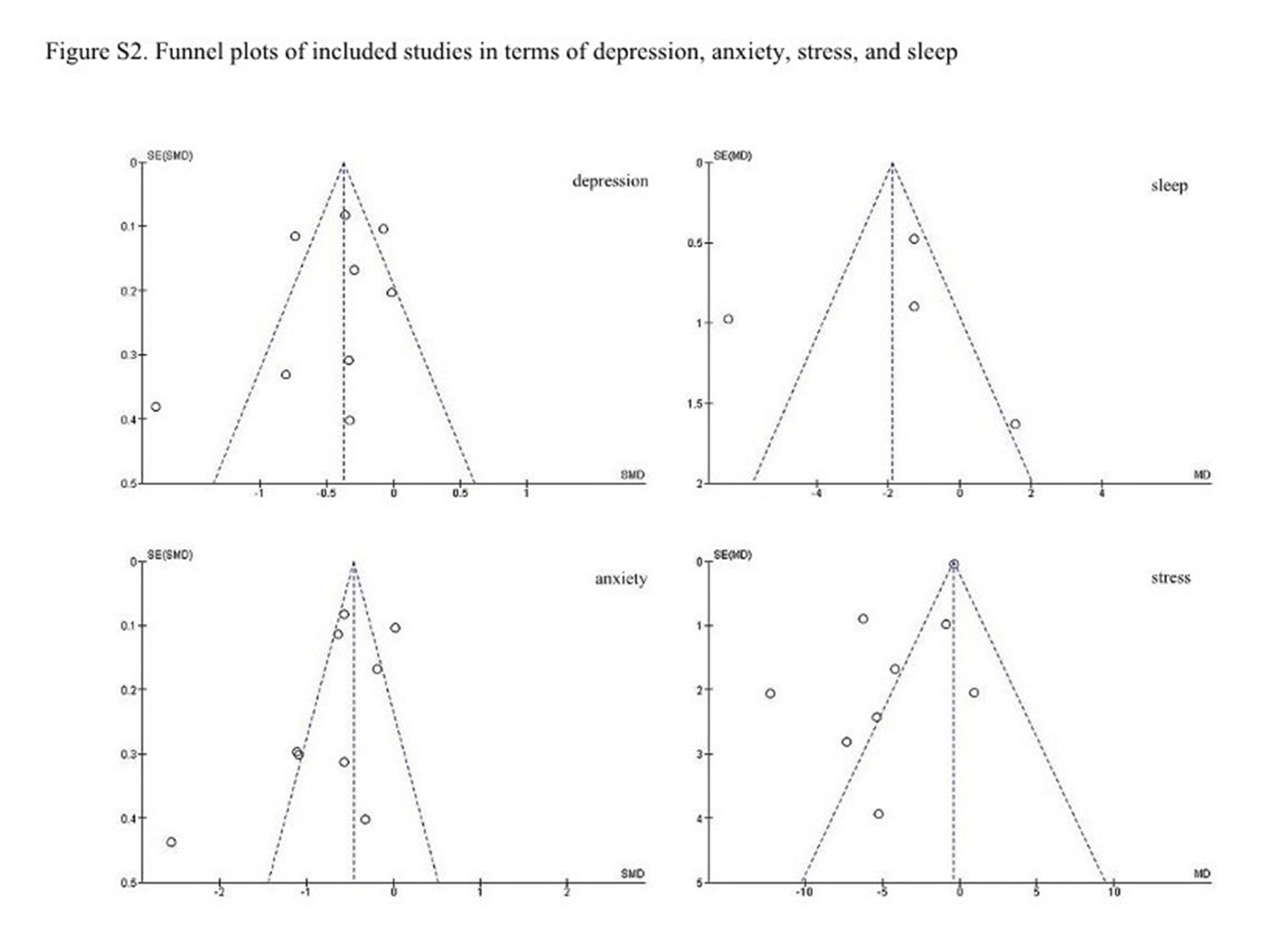

Supplement: Supplementary file 2 [file Image_2.JPEG]
